# Acute cholecystitis as a rare presentation of metastatic breast carcinoma of the Gallbladder: A case report and review of the literature

**DOI:** 10.11604/pamj.2014.17.216.3911

**Published:** 2014-03-19

**Authors:** Belhachmi Abdelilah, Ouazni Mohamed, Rajae Yamoul, Imane Elkhiyat, A Al Bouzidi, Sifeddine Alkandry, Eheirchou Abdelkader

**Affiliations:** 1 Service de Chirurgie Viscérale II Hôpital Militaire d'Instruction Med V; 2 Service de Cytologie et Anatomie Pathologique Hôpital Militaire d'Instruction Med V

**Keywords:** Acute cholecystitis, gallbladder, metastatic breast cancer, lobular breast carcinoma

## Abstract

Breast cancer is usually associated with metastases to lungs, bones and liver. Breast carcinoma metastasizing to the gallbladder is very rare. We report the case of 45-year-old female with clinical presentation of acute Cholecystitis, who underwent cholecystectomy in emergency. The Gallbladder showed a nodule on the Gallbladder wall. Histological examination disclosed a metastasis from a lobular breast carcinoma with positive hormone receptors. The patient had received three months previously a right mastectomy with axillar dissection followed by chemotherapy and radiotherapyfor lobular breast cancer stage III, PT3N1M0, showing hormone receptors. We present a rare case of acute cholecystitis from metastatic breast cancer three months after management of primary cancer.

## Introduction

Breast cancer is the most frequent malignant tumours among female. At moment of diagnosis, 60% of the patients have lymph or distant organ metastases, and about 30-80 % of the patient will develop metastatic disease following surgery and/or chemotherapy, radiotherapy or hormonotherapy [1, 2]. Breast cancer is usually associated with metastases to lungs, bones and liver. Breast carcinoma metastasizing to the gallbladder is very rare. We report a case of metastatic breast cancer that was discovered incidentally during a cholecystectomy in a patient undergoing surgery for acute cholecystitis.

## Patient and observation

**Figure 1:**
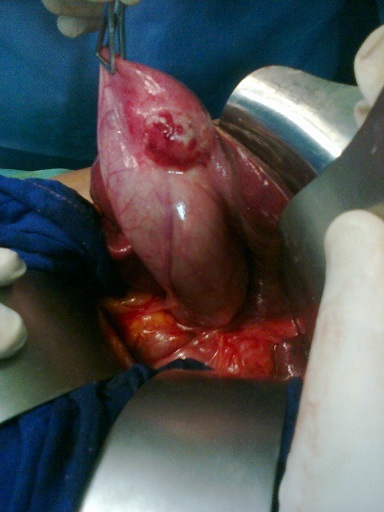
Intraoperative picture showing a nodule in the gallbladder wall

**Figure 2:**
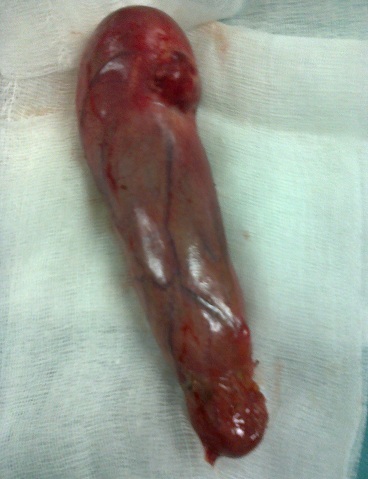
Gallbladder after resection with a nodule in the wall

**Figure 3:**
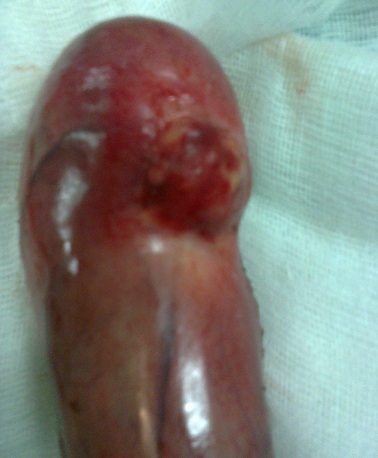
Gallbladder metastasis

**Figure 4:**
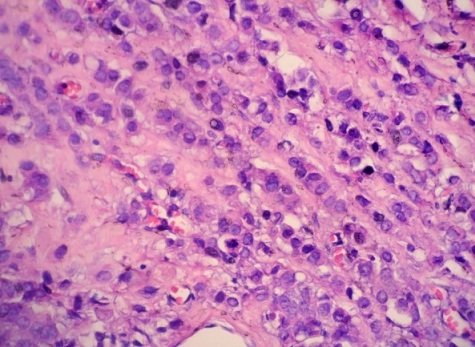
Linear or single-cell file (“Indian-file”) pattern of infiltration is characteristic of lobular carcinoma (HEx40)

**Figure 5:**
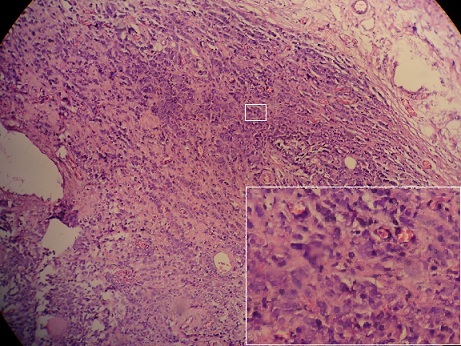
Invasion of the muscularis propria of the gallbladder by metastatic lobular carcinoma of the breast

**Figure 6:**
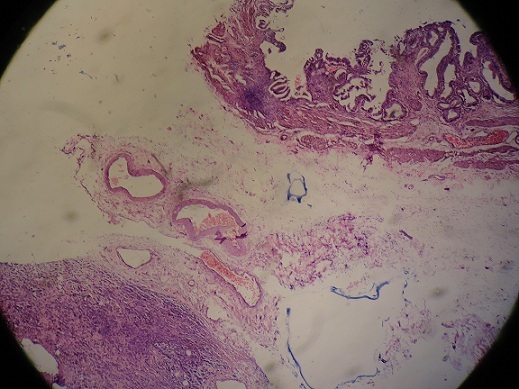
Metastatic lobular breast carcinoma (bottom left) Infiltrating the gallbladder wall (top right) : ( HEx20)

**Figure 7:**
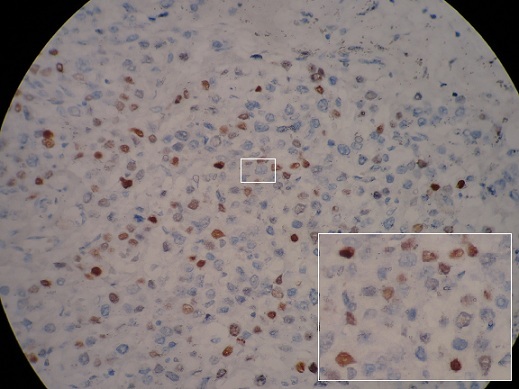
Estrogen positive cells in gallbladder wall on immunohistochemical staining

A 45-year-old woman presented with a 2-day history of progressively worsening abdominal pain in the right upper quadrant and vomiting, fever and headache. Three months previously, she had undergone a right radical mastectomy with axillar dissection, and pathologic examination revealed an invasive lobular carcinoma, corresponding to T3N1M0, Estrogen receptors, progesterone receptors and cerb B2 were positive. Adjuvant chemotherapy and radiotherapy were stared. To the emergency room, on physical examination, body temperature was 38. 3 c, blood pressure 125/65 mmHg, and pulse rate 105 /min. Abdominal examination revealed tenderness in the right upper abdomen and rigidity of the abdominal wall with positive murphy's sign. Laboratory testing revealed a hemoglobin level of 10. 5 g/dl, a white cell count of 15. 800/ul with 90 % neutrophils, Biochemical tests were within normal rang (SGPT, SGOT, ALP, gamma-GT, conjugated and unconjugated bilirubin, LDH, lipase). Ultrasound examination revealed a sludge and irregular thickness of the gallbladder. The diagnostic of acute cholecystitis was made. The patient underwent open laparotomy and cholecystectomy without complication. The nodule was perceptible and palpable on the gallbladder wall (Figure 1) , without gross involvement of the liver or regional lymph nodes, examination of the abdominal cavity showed no signs of peritoneal metastases. On gross examination, the Gallbladder was thickened, with a 1. 5 cm palpable mass (Figure 2, Figure 3, Figure 4). Histopathological examination, however, revealed the metastasis. The gallbladder wall was infiltrated (muscular layer and adventitia) by lobular breast carcinoma, hormone receptors were positive (Figure 5, Figure 6, Figure 7). Additionally, the pathological evaluation showed features of chronic cholecystitis with fibrosis of the Gallbladder wall.

## Discussion

Gallbladder is an uncommon site for metastasis. Tumours which commonly metastasize to the gallbladder are malignant melanoma and it occurs in 15 % of cases [3, 4]. Other less common primary sites leading to secondary metastasis to gallbladder include renal cell cancer, lungcancer, cervical cancer and breast cancers [5]. Breast carcinoma metastasizing to the gallbladder is extremely rare. In a large unselected autopsy series, metastases to the gallbladder were found only in 5. 8% of cancer patients [6].

Lobular cancers of the breastoften show a preference to metastasise to the gastrointestinal tract compared to ductal cancers, and very few cases of metastasis to the gallbladder were been published in the literature [7, 8]. The affinity for lobular cancers to metastasise to gastrointestinal tract is not well understood [9]. The clinical symptomatology of metastasis to the gallbladder is usually manifests with symptoms of acute/chronic cholecystitis or abdominal pain prevail [7–10]. Similarly to the reported cases symptoms of cholecystitis characterized the present case.

The differential diagnosis between primary carcinoma of the gallbladder and metastatic breast carcinoma to the gallbladder is of great importance to the management of the disease [11]. Thus, molecular biologyand immunohistochemical evaluation are often necessary.

The treatment of metastatic breast carcinoma is usually difficult, complex and sometimes aggressive, combining surgery, chemotherapy and hormonotherapy [12]. Prognosis of metastatic breast cancer is regarded as dismal and uncertain. Although resection should be considered as palliative treatment in a patient with gallbladder metastasis from breast carcinoma, it is recommended in a case which exhibits symptoms [13]. Our clinical case and review of the published cases suggests the need for careful evaluation of abdominal symptoms and strict surveillance of the gallbladder during routine imaging examination in breast cancer patients.

Although metastatic gallbladder is rare in breast cancer patients, breast cancer especially lobular type has the potential to metastasize to the gallbladder, and this diagnosis should be considered for patients with breast cancer who present with signs or symptoms of cholecystitis.

## Conclusion

Metastatic gallbladder involvement is rare, especially in a case of primary breast carcinoma. It usually manifests with abdominal pain, mimicking acute or chronic cholecystitis. The prognosis is poor [14]. Thereby, symptoms of acute/chronic cholecystitis or right hypochondrial pain in a patient with a history of breast carcinoma should raise the suspicion of metastatic gallbladder disease that must be treated properly as it portends a poor prognosis.
